# Effect of *Bifidobacterium bifidum* on Clinical Characteristics and Gut Microbiota in Attention-Deficit/Hyperactivity Disorder

**DOI:** 10.3390/jpm12020227

**Published:** 2022-02-07

**Authors:** Liang-Jen Wang, Chia-Yu Yang, Ho-Chang Kuo, Wen-Jiun Chou, Ching-Shu Tsai, Sheng-Yu Lee

**Affiliations:** 1 Department of Child and Adolescent Psychiatry, Kaohsiung Chang Gung Memorial Hospital and Chang Gung University College of Medicine, No. 123, Ta-Pei Road, Kaohsiung 83301, Taiwan; wjchou@cgmh.org.tw (W.-J.C.); jingshutsai@yahoo.com.tw (C.-S.T.); 2 Department of Microbiology and Immunology, Molecular Medicine Research Center, Chang Gung University, Taoyuan 83301, Taiwan; chiayu-yang@mail.cgu.edu.tw; 3 Division of Colon and Rectal Surgery, Chang Gung Memorial Hospital, Linkou 33332, Taiwan; 4 Department of Pediatrics, Kaohsiung Chang Gung Memorial Hospital and Chang Gung University College of Medicine, No. 123, Ta-Pei Road, Kaohsiung 83301, Taiwan; erickuo48@yahoo.com.tw; 5 Kawasaki Disease Center, Kaohsiung Chang Gung Memorial Hospital, Kaohsiung 83301, Taiwan; 6 Department of Psychiatry, Kaohsiung Veterans General Hospital, Kaohsiung 81362, Taiwan; shirleylee.ncku@gmail.com; 7 Department of Psychiatry, College of Medicine, Kaohsiung Medical University, Kaohsiung 83301, Taiwan

**Keywords:** ADHD, gut–brain axis, microbiome, probiotic, psychiatry, 16S rRNA gene

## Abstract

This study aimed to examine whether probiotics supplements using *Bifidobacterium bifidum* (Bf-688) can improve clinical characteristics and gut microbiomes among patients with attention-deficit/hyperactivity disorder (ADHD). This open-label, single-arm trial consisted of 30 children aged 4–16 years who met the criteria for ADHD diagnosis. Each subject took Bf-688, with one sachet in the morning and one in the evening (daily bacteria count 5 × 10^9^ CFUs), for 8 weeks. Patients’ clinical symptoms were assessed using the Swanson, Nolan, and Pelham Rating Scale (SNAP-IV). We collected stool samples at the baseline, the 8th week, and the 12th week for gut microbiota examination. During the 8-week Bf-688 supplement period, patients’ inattention symptoms and hyperactivity/impulsive symptoms improved, and their weights and BMIs increased. For gut microbiota, the *Firmicutes* to *Bacteroidetes* ratio (F/B ratio) decreased significantly. LEfSe analysis revealed that *Firmicutes* significantly decreased while *Proteobacteria* significantly increased during the 8-week treatment period. After Bf-688 was discontinued for 4 weeks (12 weeks from baseline), *Bacteroidota* significantly decreased and *Shigella* significantly increased. The probiotic Bf-688 supplement was associated with an improvement of clinical symptoms and with weight gain among ADHD children. Furthermore, gut microbiota composition was significantly altered by the Bf-688 supplement. A future randomized control trial is warranted to verify these findings.

## 1. Introduction

Attention-deficit/hyperactivity disorder (ADHD) is one of the most common mental disorders that occur in childhood. It affects approximately 3 to 10% school-age children around the world [1]. Children with ADHD suffer from failures in academic performance, in interpersonal relationships, and present various psychiatric comorbidities [2]. ADHD may result from multidimensional pathophysiologies [3]. A recent focal point of research has been on the multicomponent bidirectional signaling pathways between the gut and the brain [4]. The “gut–brain axis,” which refers to the link between gut microbiota and the central nervous system, has been suggested to affect neurodevelopmental disorders such as ADHD [5,6,7,8,9]. Therefore, modification of microbiota profiles by using probiotics supplements in patients with ADHD may shed light on the search for novel therapeutic applications [10,11,12,13].

One prospective study showed that supplementing a child’s diet with probiotics (*Lactobacillus rhamnosus*, LGG) during the first six months of life can reduce the risk of ADHD and autism spectrum disorders (ASD) in later childhood [14]. A clinical trial conducted in Taiwan demonstrated that *Lactobacillus plantarum* PS128 improved inattentive/hyperactive symptoms in children with ASD [15]. A randomized control trial showed that children and adolescents with ADHD who had received LGG supplementation showed better health-related QoL compared to their peers who received placebos [16]. In one study, researchers observed favorable effects of probiotic supplementation using LGG supplementation on cognitive function in children and adolescents; there was a reduced risk of developing ADHD or autism [17]. In contrast, a study in Sweden reported that both Synbiotic 2000 and placebos improved ADHD symptoms equally well. In adults with ADHD, those with elevated plasma vascular cell adhesion molecule-1 (sVCAM-1) levels proposed a reduction in autism symptoms in children and an improvement of emotional regulation [18]. In a 10-week randomized control trial for micronutrient supplementation in 17 children with ADHD, micronutrient administration modulated *Bifidobacterium* abundance and had potential implications for modulating and regulating ADHD behavior [19].

A previous case-control study revealed a nominal increase in *Bifidobacterium* genus in ADHD cases and proposed that altering the gut microbiome may result in dopamine precursor synthesis [20]. The *Bifidobacterium bifidum (B. bifidum)* species constitutes one of the dominant taxa amongst *Bifidobacterial* communities [21]. *B. bifidum* has been demonstrated to improve the quality of life in individuals with rhino-conjunctivitis seasonal allergies [22], and it has been observed to reduce milk allergies in infants [23]. Another clinical trial indicated that the *B. bifidum* TMC3115 strain (TMC3115) may exhibit beneficial effects on the serum cholesterol metabolism of subjects with dyslipidaemia via the modulation of their intestinal microbiota [24]. Furthermore, heat-inactivated *B. bifidum* MIMBb75 (SYN-HI-001) has been shown to substantially alleviate the symptoms of irritable bowel syndrome [25]. *B. bifidum* G9-1 (BBG9-1) improves dysbiosis, which results in an increase in organic acids and improved neurotransmission (including dopamine) [26]. In addition, a mixture of probiotics containing *Lactobacillus* species and *B. bifidum* could improve rotational behavior, cognitive function, and neuronal damage in patients with Parkinson’s disease (PD) [27].

Numerous studies have shown that probiotic supplementation can have a positive effect on the course of neurodevelopmental disorders, including ADHD [13]. However, the therapeutic effect of the *B. bifidum* Bf-688 strain has not yet been investigated in children with ADHD. We hypothesized that altering the dysbiosis of gut microbiota may be associated with improvements in ADHD clinical symptoms. This open-label, single-arm clinical study aimed to examine whether Bf-688 supplementation could improve the clinical symptoms of ADHD and facilitate healthy weight gain. We also investigated whether 8-week Bf-688 supplementation would change gut microbiome composition and whether any changed composition would last for more than four weeks after discontinuing Bf-688.

## 2. Material and Methods

### 2.1. Study Participants

This research protocol was approved by the Institutional Review Board (IRB) at Chang Gung Memorial Hospital in Taiwan. Eligible patients with ADHD were recruited from the outpatient Department of Child Psychiatry at Chang Gung Children’s Hospital in Taiwan. We explained the protocols of this study to participants and their parents or guardians prior to their entry into this study; written informed consent was obtained from both the children and their parents/guardians upon their agreement.

The inclusion criteria for patients with ADHD were as follows: (a) a clinical diagnosis of ADHD made by a senior child psychiatrist via structured interviews using the Diagnostic and Statistical Manual of Mental Disorders (DSM–5) [28,29]; (b) age between 4 and 16 years; and (c) no prior history of using any medical treatments for ADHD. The exclusion criteria of this study were as follows: (a) patients with a history of major neuropsychiatric diseases, including intellectual disabilities, autism spectrum disorder, bipolar disorders, major depressive disorders, psychotic disorders, or substance dependence; (b) patients with any major physical illnesses, such as epilepsy, severe head trauma, or gastrointestinal disorders; and (c) patients who are vegetarians or are currently taking any probiotics or antibiotics.

### 2.2. Study Procedure

This open-label, single-arm trial recruited 30 children aged 4–16 years who met the criteria of ADHD diagnosis. Each subject took Bf-688 for 8 weeks, with one sachet in the morning and one in the evening (daily bacteria count 5 × 10^9^ CFUs). At the baseline, 2nd, 4th, and 8th weeks for each subject, ADHD symptoms were assessed using the Swanson, Nolan, and Pelham Rating Scale (SNAP-IV) parent-form [30], and the patients’ heights and weights were measured. We collected stool samples at the baseline and at the 8th week for gut microbiota examination. In order to investigate whether the gut microbiota composition changed after discontinuing Bf-688 for four weeks, patients were also encouraged to provide fecal samples at week 12. Patients were required not to change their diet patterns; moreover, antibiotics and anti-inflammatory drugs were prohibited during the study period.

### 2.3. Sample Collection and Preparation

ADHD patients were asked to collect fecal samples by scooping a pea-sized piece of feces and placing it in a 50 mL Falcon tube. The samples were stored at 4 °C immediately after collection and then transferred to a −80 °C refrigerator in our laboratory within 24 h. From the DNA samples to the final data, including sample tests, PCR amplification as well as SMRTbell library preparation and sequencing were performed. Each step was applied to determine environmental microbial diversity and differences in microbial communities. Section 2.4, Section 2.5 and Section 2.6 describe each step of the workflow.

### 2.4. Extraction of Genome DNA, PCR Amplification, and Purification

Total genomic DNA from samples was extracted using the column-based method (i.e., QIAamp PowerFecal DNA Kit, Qiagen, Hilden, Germany). DNA concentration was determined by a Qubit 4.0 Fluorometer (Thermo Scientific, Waltham, MA, USA) and adjusted to 1 ng/ul for the process described below.

The full-length 16S gene sequence (V1–V9 regions) was amplified by barcoded 16S gene-specific primers. According to the amplification of the full-length 16S gene with barcoded primers for multiplexed SMRTbell library preparation and sequencing procedure (Pacbio), each primer was designed to contain a 5′ buffer sequence (GCATC) with a 5′ phosphate modification, a 16-base barcode, and the degenerate 16S gene-specific forward or reverse primer sequences (Forward:5′Phos/GCATC-16-base barcode-AGRGTTYGATYMTGGCTCAG-3′; Reverse: 5′Phos/GCATC-16-base barcode—RGYTACCTTGTTACGACTT-3′). The degenerate base identities were as follows: R = A, G; Y = C, T; and M = A, C. In brief, 2 ng of gDNA was used for the PCR reaction, which was carried out with KAPA HiFi HotStart ReadyMix (Roche) under the following PCR conditions: 95 °C for 3 min; 20~27 cycles (sample dependences) of: 95 °C for 30 s; 57 °C for 30 s; 72 °C for 60 s; and 72 °C for 5 min then held at 4 °C. The PCR products were monitored on a 1% agarose gel. Samples with a bright main strip of around 1500 bp were chosen and purified using AMPure PB beads for the library preparation procedure described below.

### 2.5. SMRTbell Library Construction and Sequencing

The SMRTbell library was prepared according to the amplification of full-length 16S gene with barcoded primers for multiplexed SMRTbell library preparation and sequencing procedure (Pacbio). In brief, an equal molar of each barcoded PCR product was pooled and then 500–1000 ng of the pooled amplicon sample was used for DNA damage repair followed by End Repair/A-tailing and ligation steps in order to introduce universal hairpin adapters onto the double-stranded DNA fragments. After purification with AMPure PB beads to remove adapter dimers, the SMRTbell library was incubated with sequencing primer v4 and sequel II Binding Kit 2.1 for primer annealing and polymerase binding. Finally, we performed sequencing using the circular consensus sequence (CCS) mode on a PacBio Sequel Iie instrument to generate HiFi reads with predicted accuracy (Phred Scale) = 30.

### 2.6. Data Analysis

The circular consensus sequence (CCS) reads were determined using a minimum predicted accuracy of 0.9 and a minimum number of 3 passes in the official workflow of PacBio via the SMRT Link software. After demultiplexing, the CCS reads were further processed with DADA2 in order to obtain amplicons with single-nucleotide resolutions [31,32]. The DADA2 workflow included quality filtering, dereplication, learning the dataset-specific error model, ASV inference, and chimera removal. Trimming and filtering were performed with a maximum of two expected errors per read (maxEE = 2). The DADA2 algorithm resolved exact amplicon sequence variants (ASVs) with single-nucleotide resolutions from the full-length 16S rRNA gene with a near-zero error rate. For each representative sequence, we used the feature-classifier [33] and classify-consensus-vsearch algorithm in QIIME2 [34] in order to annotate taxonomy classification based on the information retrieved from the NCBI database. In order to analyze sequence similarities among different ASVs, multiple sequence alignments were conducted by using the QIIME2 alignment MAFFT [35] against the NCBI database [36,37]. A phylogenetic tree was constructed with a set of sequences that were representative of the ASVs using the QIIME2 phylogeny FastTree [38].

Alpha diversity indicates species complexity within individual samples based on seven different criteria outputs, including the following: observed-species; Menhinick’s Richness; Margalef’s richness; Shannon index; Simpson index; PD whole tree; and good coverage. Observed-species is the number of different species represented in the microbial community. Community richness was assessed using both Menhinick’s richness and Margalef’s richness, and the relative abundance and evenness accounting for diversity were evaluated by Shannon and Simpson indices. We constructed a rarefaction curve using random selection of a certain amount of sequencing data from each sample to represent the number of observed species [39].

For statistical analysis, significance of all species among groups at various taxonomic levels was detected using differential abundance analysis with a zero-inflated Gaussian (ZIG) log-normal model as implemented in the “fitFeatureModel” function of the Bioconductor metagenomeSeq package [40]. Furthermore, Welch’s *t*-test was performed using STAMP software [41]. Statistically significant biomarkers were identified by the use of LefSe analysis [42]. In brief, LefSe is an approach based on an algorithm that performs the non-parametric Kruskal–Wallis test and Wilcoxon rank-sum test in order to identify bacterial taxa for which their relative abundance is significantly different between the control and interest groups. LefSe applies LDA to bacterial taxa identified as significantly different and further assesses the effect size of each differentially abundant taxon. In this study, taxa with an LDA score (log 10) > 4 were considered significant. Anosim and MRPP analyses were applied to determine whether community structures significantly differed among and within groups.

## 3. Results

In total, 30 children with ADHD (mean age: 6.9 years old, 80% male) were recruited for this study (Table 1). In our study sample, a high male-to-female ratio was found (4:1), which corresponds to an ADHD prevalence ratio ranging from 2:1 to 10:1 [43,44]. Among them, 23 patients completed the 8-week trial and provided fecal samples for gut microbiome analysis. Moreover, seven patients provided an additional fecal sample for analysis 4 weeks after completing the trial (12 weeks after baseline).

**Figure 1:**
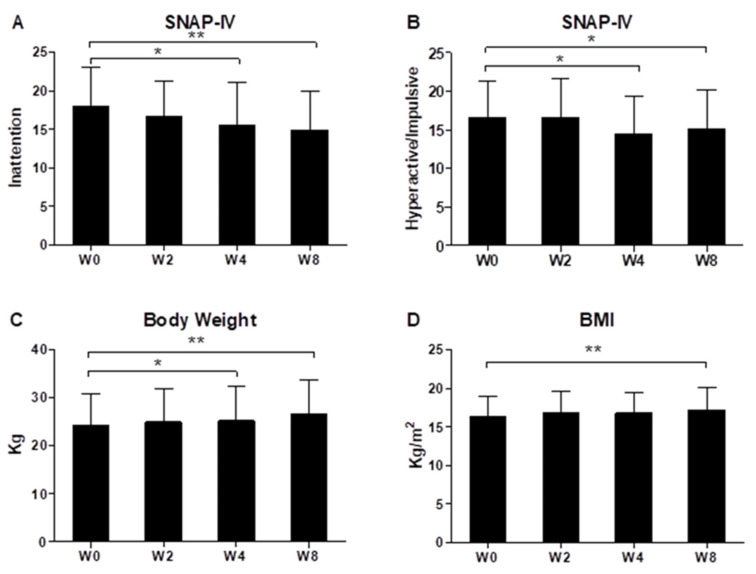
ADHD inattention symptoms (**A**), hyperactivity/impulsive symptoms (**B**), patients’ body weight (**C**) and BMI (**D**) during the eight-week period of ADHD patients receiving oral probiotic supplements (*Bifidobacterium bifidum,* Bf-688). * *p* < 0.05, ** *p* < 0.01.

As shown in Figure 1, during the eight-week period of ADHD patients receiving oral probiotic Bf-688, ADHD inattention symptoms (Figure 1A) and hyperactivity/impulsive symptoms (Figure 1B) appeared to significantly improve at both week 4 and week 8 (*p* < 0.05). During the eight-week period of ADHD patients receiving oral probiotic Bf-688, patients’ body weights (Figure 1C) significantly increased both at week 4 and at week 8, while BMI significantly increased at week 8 (Figure 1D).

With regard to gut microbiota data, sequencing depth was evaluated using a rarefaction curve to confirm the suitability of each sample. Species rarefaction curves showed a tendency toward saturation, indicating that the sequencing depth was sufficient (Figure S1A). In addition, Figure S1B shows the richness of probiotics for the different weeks. Figure S1C represents the species accumulation curve. The sequencing depth achieved included considerable information about total species richness.

**Figure 2:**
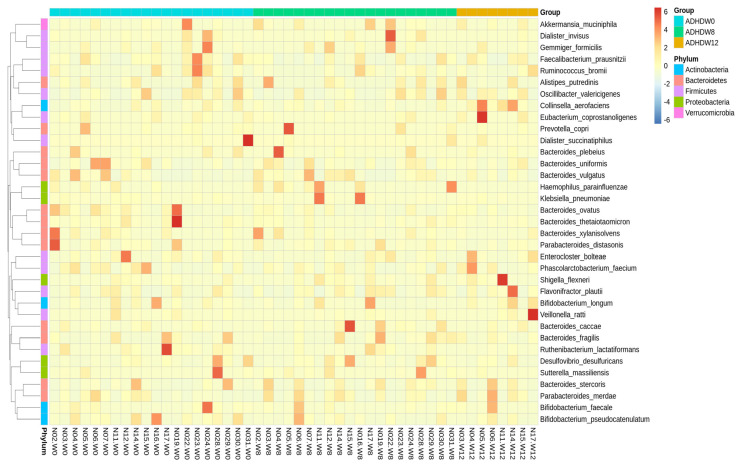
Abundance of the most prevalent bacteria at the phylum level in the fecal samples of ADHD patients at baseline, week 8, and week 12.

**Figure 3:**
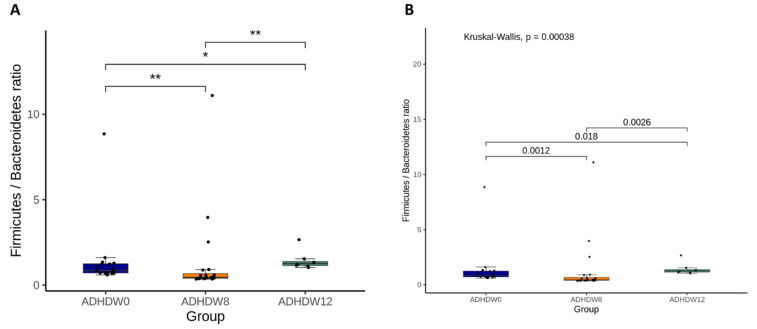
The Firmicutes to Bacteroidetes ratio (F/B ratio) at baseline, week 8, and week 12. Statistical analyses used analysis of variance (ANOVA) (**A**) and the Kruskal–Wallis test (**B**). * *p* < 0.05, ** *p* < 0.01.

Figure 2 shows the abundance of the most prevalent bacteria at the phylum level in the fecal samples of ADHD patients at the baseline, week 8, and week 12. We observed that the main phyla were *Actinobacteriota*, *Bacteroidota*, *Firmicutes*, *Proteobacteria*, and *Fusobacteriota*. The Firmicutes to Bacteroidetes ratio (F/B ratio), a relevant microbial biomarker of ADHD, significantly decreased from baseline to week 8 (*p* = 0.0012) (Figure 3). The F/B ratio increased after patients discontinued the Bf-688 supplement (from week 8 to week 12, *p* = 0.0026).

**Figure 4:**
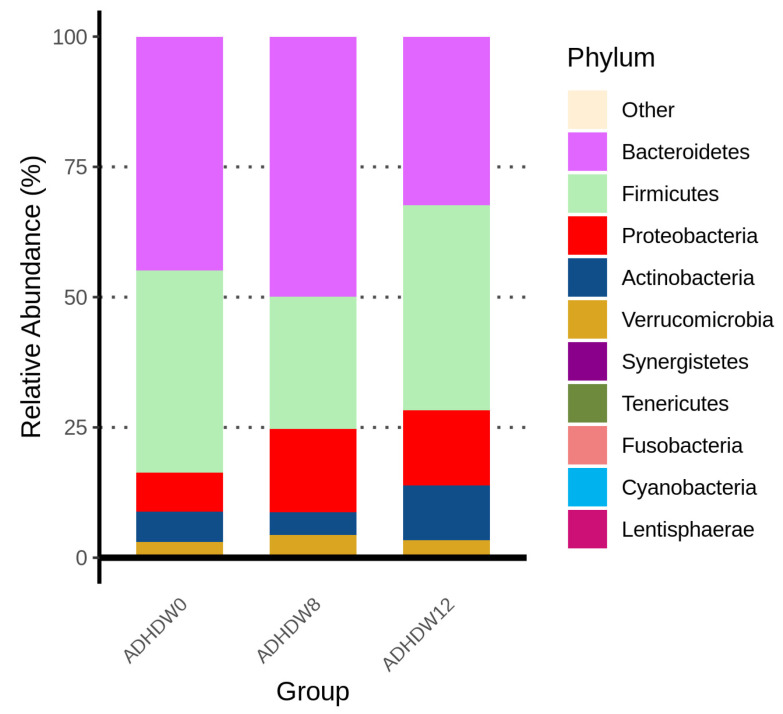
Bacterial taxonomic distributions of phylum levels in ADHD patients at baseline, week 8, and week 12.

Figure 4 illustrates the bacterial taxonomic distributions of phylum levels in ADHD patients along with time. The most abundant families are *Bacteroidota, Firmicutes,* and *Proteobacteria,* respectively. Figure S2 shows the bacterial taxonomic distributions at the species level in ADHD patients at baseline, week 8, and week 12. *Bacteroides vulgatus, Bacteroides steroids, Bacteroides uniformis, Bacteroides fragilis,* and *Shigella flexneri* were the five most abundant species among patients.

**Figure 5:**
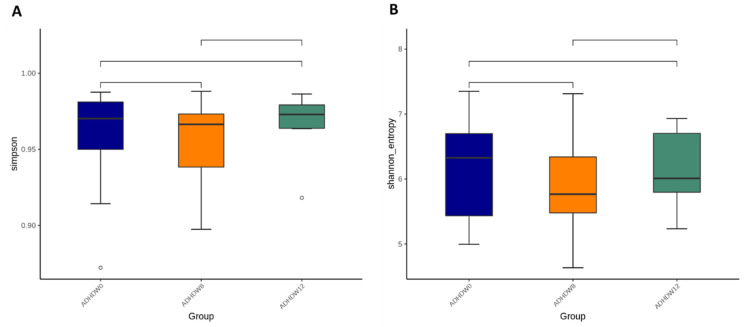
The Simpson index (**A**) and Shannon index (**B**) among ADHD patients at baseline, week 8, and week 12.

**Figure 6:**
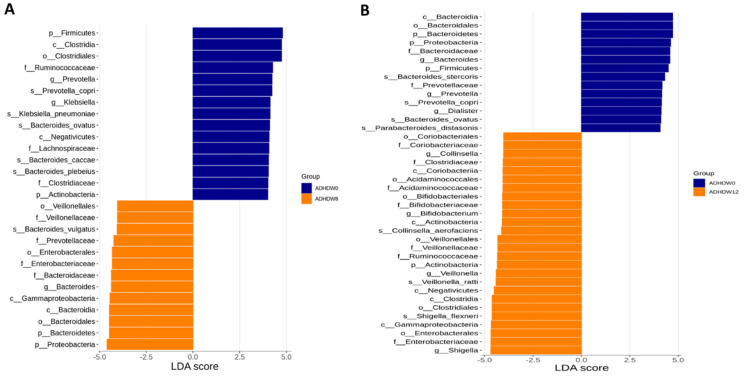
Flora with significant differences between baseline and the 8th week (**A**) and between baseline and the 12th week (**B**). The orange bars represent flora with higher abundances after taking probiotics (week 8). The blue bars show flora with higher expressions at the baseline.

We further analyzed the gut microbiota composition of ADHD subjects. The Shannon index and Simpson index were analyzed in order to determine species richness; we found no significant differences between the baseline, week 8, and week 12 (Figure 5). Figure 6 shows the flora with significant differences between the baseline and the 8th week. Figure 6A shows enriched bacteria identified at the genus level using LEfSe analysis after Bf-688 treatment for 8 weeks. *Firmicutes* significantly decreased while *Proteobacteria* significantly increased. Our previous study [45] found a high abundance of *Bacteroides ovatus (B. ovatus)* in ADHD subjects. In the current study, an 8-week probiotic (Bf-688) supplement reduced the abundance of *B. ovatus* in ADHD subjects. Figure 6B shows enriched bacteria at the genus level using LEfSe analysis after Bf-688 was discontinued for 4 weeks (12 weeks from baseline). *Bacteroidota* significantly decreased while *Shigella* significantly increased.

**Figure 7:**
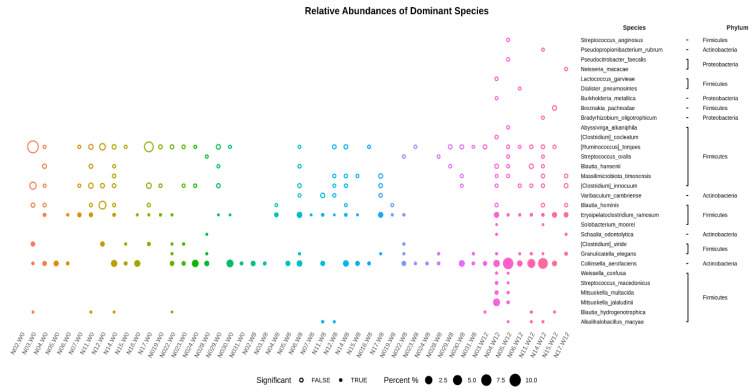
The dominant species and species abundances of each sample shown with those having abundances greater than 1% of the species (ASVs) appearing in at least one sample. The p-values between the two-group statistics (Wilcoxon rank-sum test) or the multi-group statistics (Kruskal–Wallis test). The values are sorted from small to large, and the top 30 dominant species with differences between groups are selected for the circle chart. A solid circle indicates the statistical threshold reached (*p* < 0.05), and a hollow circle represents a species under the statistical threshold.

The relative abundances of dominant species are shown in Figure 7. Abundances greater than 1% of the species (ASVs) appearing in at least one sample are illustrated. The values are sorted from small to large, and the top 30 dominant species with differences between groups were selected for the circle chart. Throughout the baseline, week 8, and week 12, *Collinsella aerofaciens* and *Erysipelatoclostridium ramosum* were collectively abundant species. However, *Clostridium viride* showed relatively high abundance in the samples at baseline (week 0). The abundances of *Granulicatella elegans* and *Blautia hydrogenotrophica* were relatively high at week 0 and decreased at week 8; then, they were re-elevated at week 12.

## 4. Discussion

This study is the first one to use *B. bifidum* (Bf-688 strain) as a supplement for ADHD, and ADHD symptoms improved during the 8-week test period. Various strains of *B. bifidum* have been demonstrated to improve several physical conditions, including rhinoconjunctivitis-specific quality of life [22], reducing milk allergies in infants [23], and improving the serum cholesterol metabolism of subjects with dyslipidemia [24], irritable bowel syndrome [25], and Parkinson’s disease (PD) [27]. *B. bifidum* G9-1 (BBG9-1) potentially improves dysbiosis, which results in modulations of organic acids and neurotransmission (including dopamine) [26]. Furthermore, neurochemical mechanisms that interfere with monoamine neurotransmitter synthesis have been implicated in ADHD pathophysiologies [46]. We proposed that Bf-688 may modulate catecholamine functions indirectly among ADHD patients. Whether ADHD symptoms improve when taking Bf-688 supplements involved in monoamine modulations still warrants further clarification.

Our clinical study revealed that ADHD patients’ body weights and BMIs significantly increased after Bf-688 administration. *Bifidobacterium* species have been proven to reduce the incidence of necrotizing enterocolitis in preterm infants [47]. A previous clinical trial found that tolerability and safety of *B. bifidum* HI-MIMBb75 was rated as very good or good by 91% of participants [25]. ADHD medications have been associated with risks of decreased appetite, weight loss, and abdominal pain in children and adolescents [48]. The results in this study imply that *Bifidobacterium* may have potential beneficial effects on gastrointestinal symptoms in ADHD patients. Whether supplements of Bf-688 improve ADHD children’s appetite or weight gain requires further validation with a randomized control trial.

Bacterial taxonomic distributions changed during the administration of Bf-688 supplements in ADHD patients. The *Firmicutes* to *Bacteroidetes* ratio (F/B ratio), a microbial biomarker, significantly decreased from baseline to week 8 and then re-elevated from week 8 to week 12. The F/B ratio evolves during different life stages [49], and a higher F/B ratio has been considered as a biomarker of obesity in animals and humans [50]. A previous clinical trial indicated that a *B. bifidum TMC3115* supplement reduced allergic scores, improved anti-inflammatory responses, and reduced the serum levels of IgE in infants. In contrast, a *B. bifidum* supplement increased the proportion of probiotics and reduced the proportion of pathogens [23]. Another study of older adults revealed that probiotics, including *B. bifidum*, maintained CD4+ lymphocytes and produced a less inflammatory cytokine profile, possibly as a result of changes in their microbial communities; furthermore, this profile more closely resembled those reported in healthy younger populations [51]. Our case–control study previously demonstrated that the abundances of *Bacteroides* and *Sutterella* may be potential gut microbiota markers of ADHD and that dietary content may be associated with gut microbiome composition [45]. Taken together with our current results, *B. bifidum* supplements changed gut microbial composition (reduced F/B ratio); however, the changed composition diminished after discontinuing *B. bifidum* supplements.

Our study has certain limitations. First, this study design was an open-label, single-arm study rather than a randomized control trial. The current study lacked a comparison group, and ADHD clinical symptoms were subjectively determined. The therapeutic effect of Bf-688 could have been easily influenced by the placebo effect. Second, the sample size was small. Only 23 patients completed the 8-week study period; thus, statistical power was limited. In addition, the age range of the participants was wide (4–16 years), and changes of gut microbiomes and behavioral patterns may be different between age groups. However, subgroup analyses stratified by age were not performed in this study as a result of the small sample size. Third, dietary habits or lifestyles may influence the gut microbiome. Although we asked patients not to change their dietary patterns and antibiotic and anti-inflammatory drugs were prohibited, the gut microbiota may still have been altered by other environmental factors. Fourth, cytokine or immunological function levels were not measured in this study. Therefore, the manner probiotics interact with gut microbiota and behavioral symptoms still requires further investigation. Additional studies are needed to clarify the molecular mechanisms that underly the gut–brain axis as the basis for modifying commensal microbiota or their functions for targeting ADHD [52].

## 5. Conclusions

The results in this study suggest oral probiotic *Bf-688* improved clinical symptoms of ADHD and increased body weights and BMIs of ADHD children. Furthermore, gut microbiota composition was significantly altered by the *Bf-688* supplement. A future randomized control trial is warranted to verify the treatment effects of probiotics (Bf-688) on ADHD symptoms.

## Figures and Tables

**Table 1:** Characteristics at baseline among ADHD patients (N = 30) who received 8-week probiotic therapy.

Variables	Mean or N	SD or %
Sex		
Boys	24	80
Girls	6	20
Age, years	6.9	1.4
Height, cm	120.6	9.3
Body weight, kg	24.2	6.7
Birth weight, g	2990.5	484.5
ADHD subtype		
Inattentive	9	
Hyperactive or combined	21	
Comorbidity ODD	7	23.3
WISC-IV, FSIQ	99.0	14.4
SNAP-IV		
Inattention	18.0	5.1
Hyperactivity/impulsivity	16.6	4.9

Notes: Data are expressed as mean ± SD or n (%); FSIQ, Full Scale Intelligence Quotient; ODD, oppositional defiant disorder; SNAP-IV, the Swanson, Nolan, and Pelham Rating Scale; WISC-IV, the Wechsler Intelligence Scale for Children—Fourth Edition.

## Data Availability

The data presented in this study are available upon request from the corresponding author.

## Supplementary Materials

The following supporting information can be downloaded at: https://www.mdpi.com/article/10.3390/jpm12020227/s1, Figure S1. The rarefaction curve of gut microbiome at baseline, week 8, and week 12. Species rarefaction curves showed a tendency toward saturation, indicating that sequencing depth was sufficient (A); the richness of probiotics for different weeks (B); the species accumulation curve (C). Figure S2. The bacterial taxonomic distributions at the species level in the ADHD patients at baseline, week 8, and week 12.
