# Supplementary figures and images for: Effect of Bifidobacterium bifidum on Clinical Characteristics and Gut Microbiota in Attention-Deficit/Hyperactivity Disorder
Source: J Pers Med. 2022 Feb 7;12(2):227. doi: 10.3390/jpm12020227 (PMC8877879; doi:10.3390/jpm12020227)

**A**

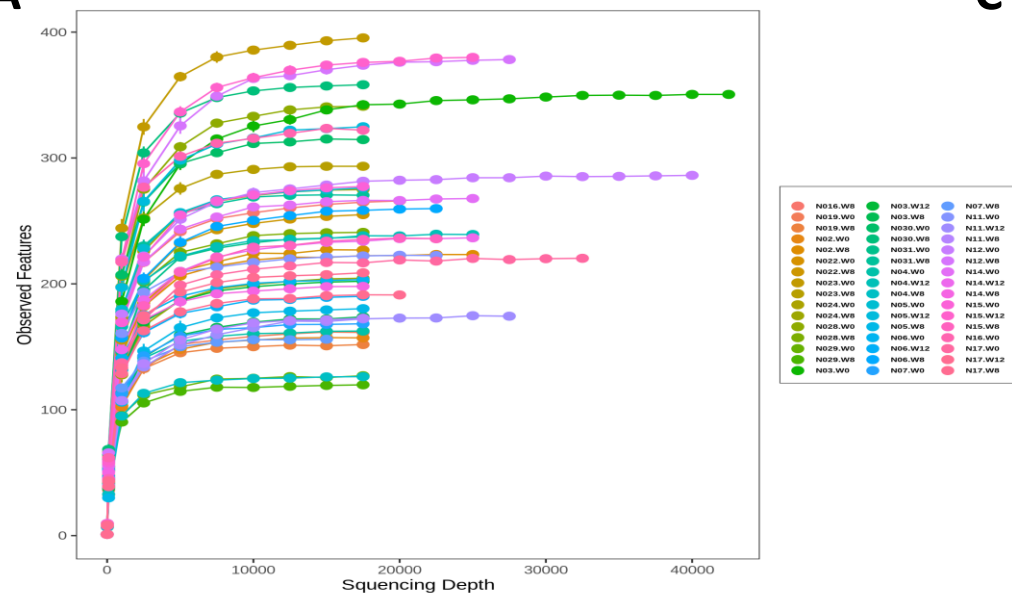

**B**

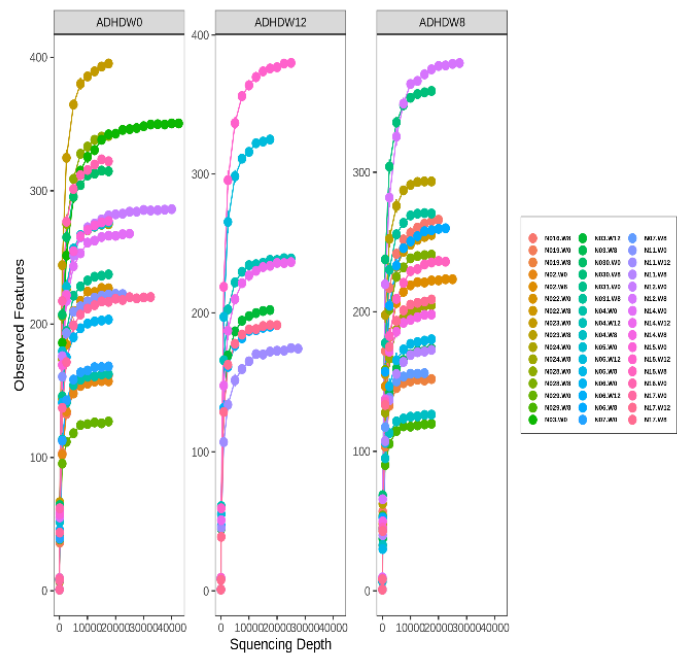

C

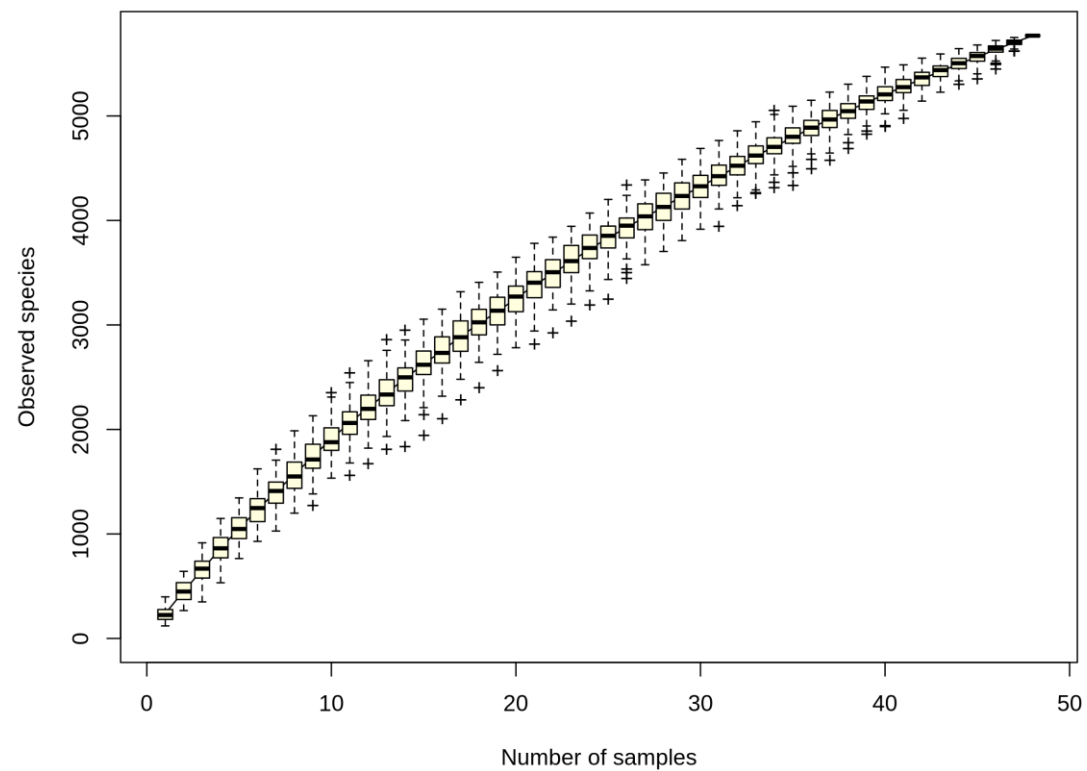

Figure S2

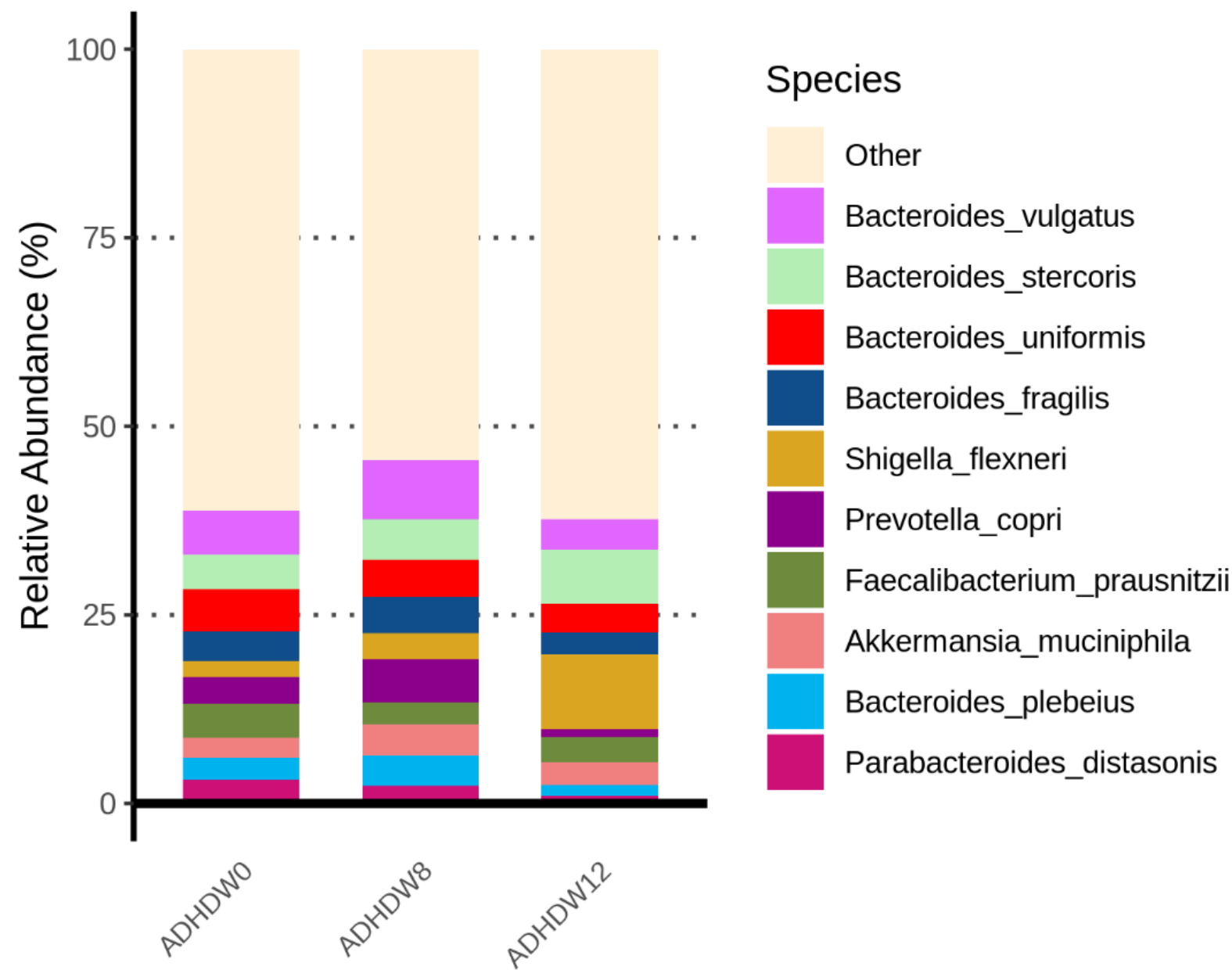

Supplement: Supplementary file 1 [file jpm-12-00227-s001.zip › jpm-1552862-SI.pdf]
